# Current Landscape of the Interrelationship Between Periodontitis, Type 2 Diabetes Mellitus, and COVID-19

**DOI:** 10.3390/ijms26188756

**Published:** 2025-09-09

**Authors:** José Luis Muñoz-Carrillo, Oscar Gutiérrez-Coronado, Paola Trinidad Villalobos-Gutiérrez, Marcelo Stalin Villacis-Valencia, Francisca Chávez-Ruvalcaba, Silverio Jafet Vázquez-Alcaraz, Oriana Rivera-Lozada, Joshuan J. Barboza

**Affiliations:** 1 Laboratorio de Inmunología, Centro Universitario de los Lagos, Universidad de Guadalajara, Lagos de Moreno 47460, Mexico; ogutierrez@culagos.udg.mx (O.G.-C.); paola.villalobos2452@academicos.udg.mx (P.T.V.-G.); 2 Facultad de Ciencias de La Salud, Escuela de Odontología, Universidad Internacional del Ecuador, Quito 170150, Ecuador; mavillacisva@uide.edu.ec; 3 Licenciatura en Nutrición, Universidad Autónoma de Zacatecas, Zacatecas 98160, Mexico; charuva@uaz.edu.mx; 4 Departamento de Odontología Conservadora, Escuela de Odontología, Universidad Complutense de Madrid, 28040 Madrid, Spain; silveriovazquez.alc@gmail.com; 5 Vicerrectorado de Investigación, Universidad Señor de Sipán, Chiclayo 14002, Peru; riveraoriana@uss.edu.pe; 6 Facultad de Medicina, Universidad Señor de Sipán, Chiclayo 14002, Peru

**Keywords:** COVID-19, periodontal disease, periodontitis, SARS-CoV-2, type 2 diabetes mellitus

## Abstract

The inflammatory response plays a central role in the pathophysiology of various chronic diseases such as periodontitis, type 2 diabetes mellitus (T2DM), and coronavirus disease 2019 (COVID-19), whose coexistence is associated with an increase in clinical complications and a more severe and serious course of these diseases. Current evidence on the interrelationship between periodontitis, T2DM, and COVID-19 remains insufficient, highlighting the need for further research to elucidate these associations. The main aim of this narrative review is to provide the current landscape of the most relevant aspects of the interrelationship between periodontitis, T2DM, and COVID-19. This narrative review was carried out through a specialized, exhaustive, and structured search of published studies indexed in the electronic databases PubMed and LILACS, for the inclusion of studies in English and Spanish, respectively, without date restriction. A search strategy was performed using the Boolean operators AND, OR, and NOT, with the following DeCS/MeSH terms: “periodontal disease”, “periodontitis”, “type 2 diabetes mellitus”, “SARS-CoV-2”, and “COVID-19”. A variety of articles were included, focusing on the most relevant aspects of the interrelationship between periodontitis, T2DM, and COVID-19. Findings suggest that inflammation is a unifying mechanism, which leads to the severity of these conditions through four shared axes: (1) a clinicopathological axis involving systemic manifestations; (2) an axis associated with metabolic alterations linked to glycemic dysregulation; (3) an axis related to enzyme overexpression linked to altered angiotensin-converting enzyme (ACE)-2 expression and glucose metabolism; and (4) an inflammatory axis. These synergistic interactions can cause these three diseases to mutually enhance each other, creating a vicious cycle, worsening the patient’s health.

## 1. Introduction

Periodontal health is defined by the absence of inflammation in periodontal tissues. Under physiological conditions, these tissues maintain an effective immune response against microorganisms present in the oral cavity, thus preserving their functional integrity [1]. Periodontal disease manifests when an imbalance occurs between immune defense mechanisms and the accumulation of subgingival biofilm, triggering an inflammatory response mediated by the innate and adaptive components of the host immune system [2]. According to the type and progression of the lesions, the development of periodontal disease presents four stages: the initial and early phases, corresponding to gingivitis, characterized by vasculitis, migration of polymorphonuclear cells (PMN) into the gingival sulcus, loss of perivascular collagen, dense infiltrate of T lymphocytes, and pathological alteration of fibroblasts; and the established and advanced stages, associated with periodontitis, characterized by a predominance of activated B cells, accompanied by increased loss of marginal gingival connective tissue matrix, PMN migration, gradual formation of gingival pockets, and destruction of alveolar bone and periodontal ligament [3]. In this context, periodontitis is a chronic disease of multifactorial etiology, characterized by an inflammatory response induced by the host to oral biofilms with dysbiotic composition. This interaction triggers a sustained immunoinflammatory process that affects the supporting periodontal tissues, causing their progressive deterioration and, in advanced stages, leading to periodontal attachment and tooth loss [4]. Diverse risk factors contribute to the onset, progression, and severity of periodontitis. These factors can be local or systemic and, and in turn, modifiable or non-modifiable. Non-modifiable factors include age, sex, ethnicity, and genetic predisposition, while modifiable factors include smoking, stress, obesity, and uncontrolled diabetes mellitus [5].

Diabetes mellitus is a metabolic syndrome with a complex etiology that encompasses a broad spectrum of genetic, epigenetic, and pathophysiological alterations. These dysfunctions can be significantly modulated by environmental factors, such as infections, diet (quality and quantity of nutrients ingested), and the composition and functionality of the gut microbiota. The interaction between these factors contributes to the development and progression of the disease [6,7,8,9]. Type 2 diabetes mellitus (T2DM) represents the most common clinical form of diabetes and is characterized by a set of interrelated biochemical and pathophysiological alterations. The main mechanisms involved include peripheral insulin resistance, inappropriate increases in hepatic glucose production, and dysfunctions in the secretion and action of intestinal hormones that modulate the homeostasis and function of insulin and glucagon, as well as a progressive decline in the functionality of pancreatic β cells. Furthermore, the presence of inflammatory processes also plays a significant role in the pathogenesis and progression of the disease [10,11,12]. In T2DM, insulin not only plays a central role in regulating energy metabolism but is also actively involved in modulating the immune response [13]. In this context, insulin resistance, a hallmark of T2DM, compromises immune homeostasis by negatively affecting the functionality of immune cells from both the innate and adaptive systems [14]. This dysfunction leads to an imbalance in the regulation of proinflammatory and anti-inflammatory responses, promoting excessive secretion of proinflammatory cytokines and adipokines, such as tumor necrosis factor (TNF)-α, interleukin (IL)-6, leptin, and resistin. These cytokines, in addition to intensifying insulin resistance, contribute to the establishment of a chronic low-grade inflammatory state. This persistent proinflammatory environment plays a key role in the progression of T2DM, by further impairing insulin signaling and compromising carbohydrate metabolism [15,16,17]. These conditions not only affect metabolism but also have significant implications for the immune response, which can promote the development of comorbidities or exacerbate other chronic inflammatory diseases, such as periodontitis. Several studies have shown a bidirectional relationship between periodontitis and T2DM [18,19], indicating that patients with T2DM are more likely to develop periodontal disease. In turn, the presence of periodontitis in patients with T2DM has been associated with poor glycemic control, suggesting a reciprocal pathological interaction that may worsen the clinical course of both conditions [20,21,22].

Coronavirus disease 2019 (COVID-19), caused by the severe acute respiratory syndrome coronavirus type 2 (SARS-CoV-2), has had an unprecedented health impact since its emergence in December 2019. Its rapid spread has led to alarming morbidity and mortality rates worldwide [23]. The clinical manifestations of COVID-19 vary widely, ranging from asymptomatic or mild symptoms to severe forms including pneumonia, acute respiratory distress syndrome (ARDS), multiorgan dysfunction, and even death [24,25]. The pathophysiology of COVID-19 is not limited to lung disorders, such as ARDS, but can affect multiple organ systems [26]. This is due to the ability of SARS-CoV-2 to infect several types of cells that express the angiotensin-converting enzyme (ACE)-2 receptor, its main entry route [27]. Susceptible cells include those of the upper respiratory tract, alveolar pneumocytes, enterocytes, endothelial cells [28], myocardial cells [29], renal tubular epithelial cells [30], and pancreatic cells [31]. This broad viral tropism produces extrapulmonary clinical manifestations with deleterious effects on multiple systems, including the neurological, thrombotic, endocrine, cardiovascular, dermatological, hepatic, renal, and gastrointestinal systems [32]. It has been documented that most patients infected with COVID-19 are asymptomatic or have mild symptoms; it is estimated that approximately 14% develop severe forms of the disease [33]. In these cases, factors such as advanced age and the presence of comorbidities, particularly T2DM [34] and periodontitis [35], have been identified as significant risk factors for disease progression toward more severe clinical presentation and even death [33]. This evidence highlights the need to further analyze the interactions between these pathologies, with the purpose of understanding their combined influence on the clinical course of the disease and improving comprehensive management strategies for patients with these comorbidities. Currently, the available scientific evidence on the interrelationship between periodontitis, T2DM, and COVID-19 remains limited. Therefore, a broader and more rigorous literature search is needed to identify additional studies, and in this way provide an updated view on the association of these three pathologies. In this context, this narrative review aims to provide an updated and comprehensive landscape of the main aspects that define the interrelationship between these pathologies, emphasizing shared pathophysiological mechanisms, clinical implications, and their impact on outcome severity.

## 2. Materials and Methods

**Figure 1:**
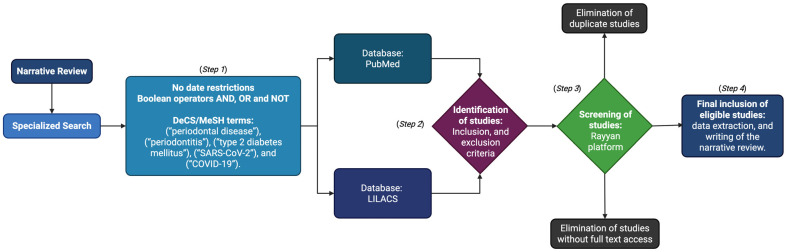
Flowchart of the methodology applied in the narrative review. Step 1: Specialized search. Step 2: Identification of studies. Step 3: Screening of studies. Step 4: Inclusion and analysis of studies. Figure created with BioRender (https://www.biorender.com) by Muñoz-Carrillo et al.

This study involved a narrative review, which was carried out according to the following methodology [36] in four steps (Figure 1).

### 2.1. Step 1: Specialized Search

An exhaustive and structured search was carried out in the electronic databases PubMed and LILACS, using Boolean operators (AND and/or OR) and DeCS/MeSH controlled terms such as “periodontal disease”, “periodontitis”, “type 2 diabetes mellitus”, “SARS-CoV-2” and “COVID-19”.

### 2.2. Step 2: Identification of Studies

Studies were selected based on previously established eligibility criteria, which considered thematic relevance, quality, and scope for this study. Inclusion criteria were as follows: (1) articles in English or Spanish; (2) no publication date restrictions; (3) studies of the following type or design: clinical trials, observational studies, relevant narrative reviews, systematic reviews, clinical cases; (4) studies addressing comorbidity between periodontitis and T2DM; (5) studies analyzing the relationship between T2DM and COVID-19; (6) studies exploring the association between periodontitis and COVID-19; (7) articles explicitly mentioning the interrelationship between the three pathologies: periodontitis, T2DM, and COVID-19. Exclusion criteria were as follows: (1) studies that do not directly address any of the comorbidities; (2) studies of the following type or design: conference abstracts and letters to the editor.

### 2.3. Step 3: Screening of Studies

During this step, duplicate studies were identified and eliminated. Articles were then selected, applying eligibility criteria based on their title and abstract, ensuring their relevance and quality. Studies without access to the full text were eliminated. To facilitate this process, the Rayyan platform was used https://www.rayyan.ai (accessed on 15 April 2025).

### 2.4. Step 4: Inclusion and Analysis of Studies

Finally, the studies that had passed the preliminary steps and met all eligibility criteria were retrieved and analyzed in full text. Relevant data were then extracted, and this narrative review was written, integrating the most significant findings regarding the association between periodontitis, T2DM, and COVID-19.

## 3. Periodontitis and Type 2 Diabetes Mellitus

**Figure 2:**
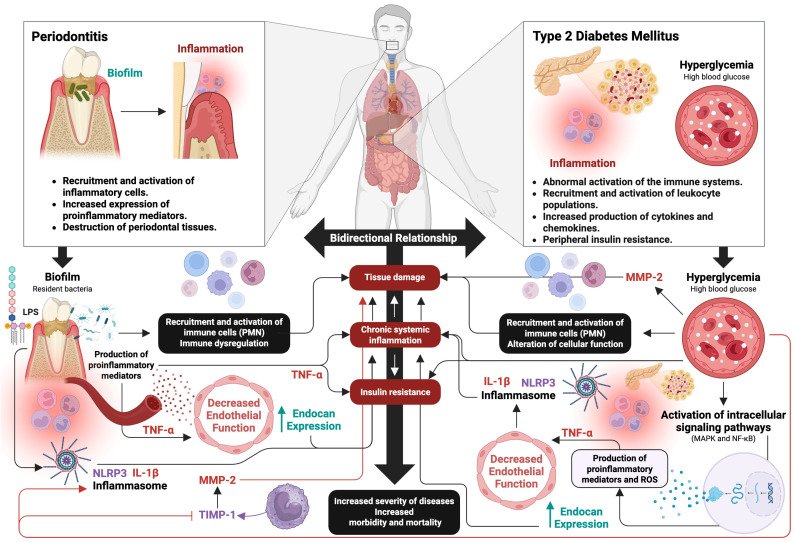
Immunopathological mechanisms in the interrelation between periodontitis and T2DM. The figure represents the bidirectional interrelation between periodontitis and T2DM. The exacerbated inflammatory response in both conditions promotes the overexpression of proinflammatory cytokines, such as TNF-α and IL-1β, which induce insulin resistance, sustained hyperglycemia, and endothelial dysfunction. TNF-α also promotes an increase in endocan, a marker of endothelial activation, exacerbating vascular inflammation. In the periodontal environment, these processes promote the activation of MMP-2, leading to the degradation of periodontal tissues. Furthermore, induction of cell apoptosis and functional inhibition of PMN are observed, compromising the local immune response. These mechanisms generate a vicious cycle that enhances the progression of both diseases. Figure created with BioRender (https://www.biorender.com) by Muñoz-Carrillo et al.

Periodontitis has been recognized as the sixth complication of T2DM, due to solid scientific evidence supporting a bidirectional relationship between the two pathologies [37]. Several studies have reported that patients with T2DM (whether controlled or uncontrolled) have a higher prevalence of chronic or severe periodontitis compared to healthy individuals [38,39,40,41,42,43]. This reciprocal interaction is explained, in part, by the presence of pathogenetic inflammatory mechanisms common to both diseases (Figure 2). To compare the inflammatory mechanisms between periodontitis and T2DM, it is necessary to describe how these diseases affect each other.

T2DM is characterized by dysregulation of the innate and adaptive immune responses, even without the presence of external pathogens or antigens [44,45]. This dysregulated immune response significantly affects the metabolic system. Elevated blood glucose levels, or hyperglycemia, a hallmark of T2DM, contribute to the development and progression of inflammation and disease [46]. Hyperglycemia in T2DM favors the increase of PMN within the affected tissues [47,48,49], altering several functions such as cell adhesion, chemotaxis, phagocytosis, and antigen degradation, causing tissue damage by these cells [50,51]. In turn, it has been observed that, during T2DM, hyperglycemia is associated with PMN deficiency in the processes of healing and tissue repair during inflammation [52], in addition to presenting defects in apoptosis, causing a decrease in functional longevity and an increase in the elimination of PMN in infectious sites, contributing to greater susceptibility to and severity of infections in diabetic patients [53]. Furthermore, during periodontitis, bacteria in the gingival sulcus stimulate the activation of PMN, which increases the secretion of bactericidal molecules. These molecules play a key role in the progression of periodontal disease, contributing to the destruction of periodontal tissue [54] and the development of inflammation, which may contribute to metabolic dysregulation [55].

Current evidence indicates that elevated PMN levels in periodontitis patients show immune dysregulation, which plays a crucial role in the etiology of the disease [56]. In this sense, T2DM is considered a significant risk factor for the development and severity of periodontitis and vice versa [57,58]. This is because both diseases share a complex relationship involving hyperinflammation, especially caused by hyperreactive PMN [55], since the different phenotypes presented by PMN act as an important link in both diseases, influencing their pathogenesis. Therefore, PMN acts as a pathogenic link capable of influencing comorbidities. In this context, studies confirm these findings, since, on the one hand, it has been observed that patients with periodontitis and T2DM show a significant increase in gingival PMN, compared to individuals with periodontitis only, indicating a hyperinflammatory reaction in the gingival tissue, probably due to T2DM [59], which suggests that inflammation may be a bilateral factor that can increase the severity and progression of both diseases [60]. On the other hand, an alteration of PMN apoptosis in peripheral blood has been observed in individuals with T2DM. Furthermore, periodontitis acted as a confounding factor, meaning that it exerted an additive effect, significantly delaying spontaneous PMN apoptosis in patients with T2DM and periodontitis. These findings suggest that periodontitis not only affects PMN apoptosis at the site of periodontal infection but also has a systemic impact on the resolution of inflammation and PMN clearance. This may contribute to the exacerbation of other systemic inflammatory diseases, such as T2DM. Indeed, PMN apoptosis has been shown to be delayed in periodontal disease due to the action of TNF-α [61].

Hyperglycemia also contributes to the development and evolution of inflammation during T2DM, through the activation of various intracellular signaling pathways, such as the mitogen-activated protein kinase (MAPK) and nuclear factor (NF)-κB pathways, resulting in an increase in the production of proinflammatory mediators, such as cytokines and reactive oxygen species [62,63,64,65]. Likewise, periodontitis can influence the development and state of chronic systemic inflammation, through the aberrant increase in proinflammatory cytokines [66], affecting endothelial function, and substantially contributing to the development of insulin resistance, causing a homeostatic imbalance in blood glucose regulation [67,68,69]. In this context, T2DM intensifies the inflammatory response in periodontal tissues [70], leading to an increase in the expression of inducible nitric oxide synthase (iNOS) and in the levels of lipid peroxides [71] and proinflammatory mediators, such as prostaglandin (PG)-E_2_, TNF-α, IL-1β, IL-6, IL-17, and, IL-23, in periodontal tissues and in gingival crevicular fluid (GCF) [72], which contributes to a more severe course of periodontal inflammation [73,74,75]. This is because the exacerbated production of proinflammatory mediators influences the vascular and cellular phenomena of inflammation [76], stimulating bone resorption, through an increase and reduction in the expression of the receptor activator of nuclear factor-κB ligand (RANKL) and osteoprotegerin, respectively [77]. In addition, a decrease in the production of anti-inflammatory lipid mediators and cytokines such as IL-4, IL-10, and transforming growth factor (TGF)-β has been reported, which potentially contributes to the development and aggravation of periodontal inflammation in patients with T2DM [66,78].

Among these proinflammatory mediators, TNF-α, an important pleiotropic proinflammatory cytokine [79], plays an important role in T2DM [80], as it can exert direct effects on vascular endothelial cells to induce endothelial dysfunction and subsequent damage to vascular function [81]. Endothelial dysfunction produces functional alterations to endothelial cells and is mainly characterized by the suppression of the release and bioavailability of nitric oxide (NO), in addition to the increased expression of adhesion molecules and chemokines [82]. The interaction between these alterations and the smooth muscle cells located in the blood vessels in turn alters vascular structure and function [83]. T2DM is closely related to vascular endothelial dysfunction, affecting the protective balance and permeability of the endothelium and enhancing chronic systemic inflammation [84,85]. In this context, TNF-α plays a key role in regulating the expression of endocan, a soluble proteoglycan whose production is markedly increased in the vascular endothelium in response to endothelial activation and systemic inflammation [86]. This dual functionality allows endocan to act both as an inflammatory mediator and as a biomarker of endothelial activation [87]. Interestingly, a correlation has been observed between increased endocan levels and impaired glycemic control; in contrast, improved glycemic control is associated with decreased expression of this biomarker [88]. Likewise, endocan expression has been detected in systemically healthy individuals with periodontal disease who do not present systemic alterations [89], suggesting a possible implication beyond glycemic control. In this context, endocan is postulated as a promising biomarker for the early diagnosis and prognosis of chronic inflammatory states, both in type 2 diabetes mellitus and periodontitis, due to its ability to reflect the endothelial activation associated with these conditions. Additionally, its measurement could be useful as an indicator of therapeutic monitoring in patients with T2DM and periodontal disease, given that its levels are correlated with the systemic inflammatory state and glycemic control in these individuals [90].

The NOD-like receptor (NLR) family pyrin domain containing 3 (NLRP)-3 inflammasome, a supramolecular complex [91], plays an important role during the inflammatory response [92], such as in T2DM and periodontitis. An association between vascular dysfunction in T2DM and NLRP3 inflammasome activation has been reported [93]. Likewise, an increase in the expression and activation of NLRP3 has been demonstrated in the presence of periodontal inflammation, playing an important role in the inflammatory response [94]. Therefore, T2DM may increase NLRP3 expression in patients with chronic periodontitis, with increased IL-1β production [95]. In turn, periodontitis, in patients with T2DM, contributes, through its chronic inflammatory effects, to greater positive regulation of NLRP3 concentrations, both at the salivary and systemic levels [96]. In this context, several studies have reported elevated expression of the NLRP3 inflammasome, both in the gingival tissues of patients with periodontitis [97,98] and in cells of the innate immune system and pancreatic β-cells in patients with T2DM [99,100]. In addition, it has been documented that, both in patients with chronic periodontitis and T2DM, and in human gingival epithelial cells (HGEC) exposed to lipopolysaccharide (LPS) and high glucose concentrations, a significant increase in the expression of the NLRP3 inflammasome and IL-1β is observed. These findings indicate that hyperglycemia may intensify the inflammatory response in gingival tissues through the NLRP3 pathway, favoring greater tissue degradation [101]. This is because elevated levels of IL-1β have been significantly associated with the immunopathology of periodontitis, promoting the destruction of periodontal tissue, especially through alveolar bone resorption and damage to the lamina propria [102].

On the other hand, hyperglycemia and inflammation induce an increase in the production and activation of matrix metalloproteinases (MMPs), which leads to the destruction of connective tissue and induction of apoptosis in fibroblasts and osteoblasts, thus limiting the repair capacity of periodontal tissues [103,104]. MMPs are essential enzymes involved in tissue remodeling and extracellular matrix (ECM) degradation [105]. They are also involved in the functional regulation of various biologically active substrates [106], such as pro- and anti-inflammatory cytokines, chemokines, growth factors, serum components, components of the complement system, and molecules involved in cell signaling, all of which play a key role in modulating the immune response [107]. During T2DM, hyperglycemia induces an increase in MMP levels [108], among which is MMP-2 [109,110]. Likewise, MMP-2 is a highly active MMP present in saliva and GCF, which plays a crucial role in the degradation of periodontal tissues [111,112,113]. Several studies have demonstrated an association between MMP-2 and periodontitis, given that its activity is regulated by tissue inhibitors of matrix metalloproteinases (TIMPs) [114], particularly TIMP-1, considered its main endogenous inhibitor. This regulator is produced by periodontal cells, macrophages, and monocytes [115]. In this context, a significant increase in MMP-2 enzymatic activity as well as TMP-1 expression has been observed, according to the severity of periodontitis. In addition, a significant increase in glycosylated hemoglobin (HbA1c) levels has been found in patients with moderate and severe periodontitis, suggesting that poor glycemic control is associated with periodontitis severity. Furthermore, patients with poor glycemic control have been shown to have a significant increase in PMN, accompanied by a marked decrease in MMP-2 and TIMP-1 activity. These findings indicate that an imbalance in MMP-2/TIMP-1 occurs in individuals with T2DM and poor glycemic control, and that the TIMP-1-mediated mechanism of MMP-2 activity inhibition is compromised in severe periodontitis [116].

## 4. Periodontitis and COVID-19

At the clinical level, several studies have evidenced an association between periodontitis and adverse outcomes of COVID-19 [117,118,119,120], as patients with periodontal disease have been shown to be at increased risk of severe COVID-19 [121,122], including hospitalization, assisted ventilation, intensive care unit admission, and higher mortality rate [123,124,125], mainly through some possible mechanisms, including local and systemic inflammatory responses [126,127,128]. Conversely, COVID-19 can exacerbate periodontal disease, leading to increased gingival bleeding, dental plaque accumulation, and deepening of periodontal pockets [129,130]. This exacerbation is associated with microbial dysbiosis, bacterial superinfections, host hypersensitivity, and overstimulation of the immune system, resulting from the complex interaction between the environmental, microbial, and inflammatory factors that promote the progression of the disease [131].

**Figure 3:**
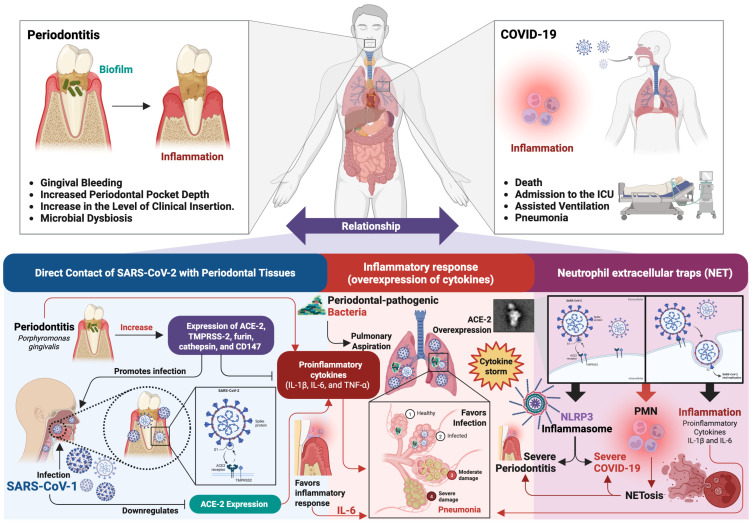
Interrelation between periodontitis and COVID-19. The figure illustrates three main mechanisms that explain the relationship between periodontitis and COVID-19: First mechanism: direct entry of SARS-CoV-2 into periodontal tissues through elevated expression of ACE-2. Second mechanism: activation of a shared systemic inflammatory response, with overproduction of proinflammatory cytokines that can lead to a cytokine storm. Third mechanism: hyperreactivity of PMN with the formation of NET, contributing to tissue damage and progression of periodontal disease. Figure created with BioRender (https://www.biorender.com) by Muñoz-Carrillo et al.

According to current scientific evidence, three main and interrelated mechanisms could explain the association between periodontitis and COVID-19 disease (Figure 3). The first mechanism refers to direct viral infection of periodontal tissues, favored by the elevated expression of the ACE-2 in these tissues [132]. The second mechanism involves a common inflammatory response, characterized by the overexpression of proinflammatory cytokines, which can trigger a cytokine storm, a phenomenon associated with severe forms of COVID-19 [133]. The third mechanism is related to the hyperreactivity of PMN and the formation of neutrophil extracellular traps (NET), events that significantly contribute to tissue damage and the destruction of periodontal structures [134].

*Direct contact of SARS-CoV-2 with periodontal tissues*. In periodontitis, periodontal pockets provide favorable environments for the replication of pathogenic viruses [135], which can enter systemic circulation through the GCF and then mix with saliva. Therefore, periodontal pocket epithelium [136,137], GCF [138], and saliva [139] represent important sources of viral particles and potential modes of virus transmission [140]. In this context, periodontitis-induced ulceration of the gingival epithelium may compromise its protective function, increasing the risk of SARS-CoV-2 invasion [141], since SARS-CoV-2 is capable of infecting and replicating directly in oral structures, including periodontal tissues [142,143]. The main receptor in human cells that allows the entry of SARS-CoV-2 is ACE-2. ACE-2 is expressed in a variety of tissues, including the lungs, nasopharyngeal mucosa, salivary glands, and oral mucosa [144]. Within the oral mucosa, ACE-2 is mainly expressed in oral epithelial cells of the tongue, fibroblasts, gingival tissues, periodontal pockets, and gingival crevices [145]. At the level of the oral cavity, ACE-2 expression is higher in the salivary glands compared to the lung, which act as an important reservoir of SARS-CoV-2, facilitating efficient infection of the virus [146]. In addition to the expression of ACE-2, other molecules have been identified whose expression is necessary to allow SARS-CoV-2 infection [147]. These molecules include transmembrane serine protease (TMPRSS)-2, furin, cathepsin, and cluster of differentiation (CD)-147 [148,149,150,151]. Studies have reported that, in the oral cavity, there is high expression of these molecules, mainly in the epithelial cells of the oral mucosa, in gingival keratinocytes, in the periodontal tissues and pockets, and in the GCF [139,145,152,153]. Therefore, co-expression of these molecules is necessary to activate the S protein of SARS-CoV-2 and thus bind to host cells and further increase SARS-CoV-2 infectivity in the oral cavity [154].

On the other hand, the regulation of the expression of these molecules plays a fundamental role in the pathogenesis of the disease. In the oral cavity, periodontal–pathogenic bacteria, such as *Porphyromonas gingivalis*, are capable of inducing an increase in the expression of ACE-2, TMPRSS-2, and furin in resident cells, such as fibroblasts [155]. High expression of ACE-2 downregulates the production of proinflammatory cytokines, such as IL-1β, IL-6, and TNF-α [145]. However, this high expression of ACE-2 favors the entry of SARS-CoV-2 into the oral cavity [156]. In this sense, infection and replication of SARS-CoV-2 downregulate ACE-2 expression, leading to an increase in proinflammatory cytokines, favoring the inflammatory response [157]. These findings suggest that local inflammation promotes the spread of SARS-CoV-2 infection and replication in periodontal tissues, with possible further systemic expansion. On the other hand, aspiration of periodontal–pathogenic bacteria could increase the risk of SARS-CoV-2 infection, since they can increase the expression of ACE-2 in the lungs and bronchi [158], and induce the production of proinflammatory cytokines [159], such as IL-6, by alveolar and bronchial epithelial cells, which promotes SARS-CoV-2 infection, and lower respiratory tract inflammation can become severe in the presence of viral pneumonia, contributing to the development of cytokine storm and acute respiratory distress syndrome [160].

*Inflammatory response (overexpression of cytokines)*. During periodontitis, the presence of bacteria in the subgingival biofilm produces a chronic inflammatory response, which is mainly characterized by an overproduction of cytokines, known as cytokine storm, which is related to the degradation and destruction of periodontal tissues [161]. Likewise, COVID-19 also has the ability to trigger a cytokine storm, causing tissue damage, especially in the connective tissue of the lungs [162]. In this regard, much higher levels of serum markers of systemic inflammation have been found in patients with COVID-19 and periodontitis [163,164]. One of the cytokines that plays an important role in both diseases is IL-6, since studies have demonstrated the strong immunopathogenic role of this proinflammatory cytokine in the rational and progressive destruction of these two conditions [165]. In periodontitis, IL-6 is also an overexpressed cytokine, which is associated with tissue damage [166], as patients with periodontitis have been reported to have significantly higher levels of IL-6 in saliva and serum, associated with periodontitis severity and tooth loss [167,168,169]. Meanwhile, in COVID-19, overexpression of IL-6 in the lungs causes interstitial pneumonia, multiorgan damage, and risk of death [162]. Serum IL-6 levels have been correlated with the stage of COVID-19 disease, particularly in patients experiencing respiratory failure [170]. Therefore, elevated IL-6 levels can be used as a predictive biomarker to identify patients at risk of disease progression [171]. Furthermore, increased expression of the IL-6 receptor (IL-6R) and higher levels of IL-6 have been observed in COVID-19 patients who did not survive compared to patients who survived throughout the clinical course of the disease [33]. These findings suggest a potential role of IL-6 in the pathogenesis and progression of COVID-19 [172].

The NLRP3 inflammasome plays a key role in the cytokine storm observed during both COVID-19 and periodontitis [173], since its activation induces the synthesis and release of proinflammatory cytokines, which causes tissue damage and inflammation in both diseases [174]. NF-κB induces the transcriptional expression of NLRP3 and pro-IL-1β [175,176]. Activation of the NLRP3 inflammasome results in the release of proinflammatory cytokines IL-1β and IL-18 [177], thereby promoting inflammation and other associated disorders. Inflammatory cytokines can promote the development of low-grade systemic inflammation, leading to the abnormal activation of the NLRP3 inflammasome. This, in turn, can drive chronic inflammatory conditions and influence the pathophysiology of inflammation-related diseases [178]. Patients with periodontitis have been observed to have significantly higher levels of NLRP3, both in blood and saliva. In this sense, NLRP3 inflammasome-related proteins, such as IL-1β, have been proposed as potential biomarkers of periodontal clinical status [172]. Studies have reported that the expression of these proteins is associated with alveolar bone loss and periodontal tissue destruction [179], a hallmark of periodontal disease, and an increase in proinflammatory cytokines, which can contribute to the severity of periodontal disease [180]. The severity of COVID-19 with tissue damage and relevant cytokine storm has been correlated with NLRP3 inflammasome activation [181,182]. Post-mortem analysis of COVID-19 patients has revealed persistent NLRP3 inflammasome activation in various tissues and PMN from peripheral blood [172]. This is because, after viral replication, ACE-2 decreases its activity, activating ACE-1, leading to elevated levels of PMN, reactive oxygen species, NF-κB, and proinflammatory cytokines, ultimately resulting in inflammatory cell death and tissue damage [162]. The NLRP3 inflammasome has been observed to be increased in serum and saliva in patients with periodontitis, playing an important role in the COVID-19 cytokine storm [183], and has been shown to aggregate in the lungs, resulting in fatal pneumonia [184], thus providing the potential molecular link between viral infection and cytokine release syndrome [185].

*Neutrophil extracellular traps (NET).* PMN hyperreactivity is a hallmark of both periodontitis [186,187] and COVID-19 [188] which contributes to the strong inflammatory response, such that, instead of exerting a protective action, PMN promotes tissue destruction in the host [189]. One of the main actions of PMN is the release of NET, which works through mechanical entrapment of pathogens or other insults [190]. However, in some clinical conditions, the generation of NET (NETosis) leads to host cell damage and death at the tissue level, directly or indirectly through immune mechanisms, developing a state of severe inflammation [191]. Currently, there is increasing evidence of the immunopathological role of PMN in the development of periodontitis and severe forms of COVID-19 [192]. NETosis plays a potential role in the pathogenesis of periodontitis [193]. Studies have reported an abundance of NET from PMN and some phagocytic PMN in GCF [194] and periodontal tissues [195], which are associated with the severity of periodontitis [196]. On the other hand, a correlation has been shown between COVID-19 severity and PMN count and activation [191]. Hospitalized COVID-19 patients have been reported to have increased PMN in the bloodstream and lungs, which exhibit an activated phenotype with enhanced NETosis and oxidative burst [188]. SARS-CoV-2 is capable of activating NETosis in human PMN [197,198], which plays an important role in the pathophysiology of inflammation and coagulopathy, generating organic damage through immunothrombosis, which characterizes severe cases of COVID-19 [199]. In this context, PMN hyperreactivity and NET formation are important factors in the pathogenesis of both diseases. Therefore, patients with periodontitis may be at higher risk of developing a more severe course of COVID-19, as PMN and NET formation could increase the destruction of compromised tissues and consequently increase the risk of mortality [192].

## 5. Type 2 Diabetes Mellitus and COVID-19

Patients with T2DM have increased susceptibility to several common infections [200], attributed to a dysfunction of innate immunity and inappropriate inflammatory responses [201]. In this context, current clinical evidence reveals that comorbidity between T2DM and COVID-19 makes these two diseases mutually promoting and risk factors for each other [202,203]. Studies have reported that patients suffering from this comorbidity of T2DM and COVID-19 present more severe respiratory symptoms, with a higher incidence of acute complications, worsening renal function [204], and worse clinical outcomes, presenting a severe inflammatory response, severe pneumonia, and multiorgan damage, with a higher risk of admission to the intensive care unit, receiving mechanical ventilation, and in-hospital mortality [205,206,207,208]. In this sense, it has been reported that hospitalized patients with T2DM and COVID-19 have almost double the risk of mortality [209,210], with a prevalence of death between 22 to 31%, compared to patients without T2DM [211].

**Figure 4:**
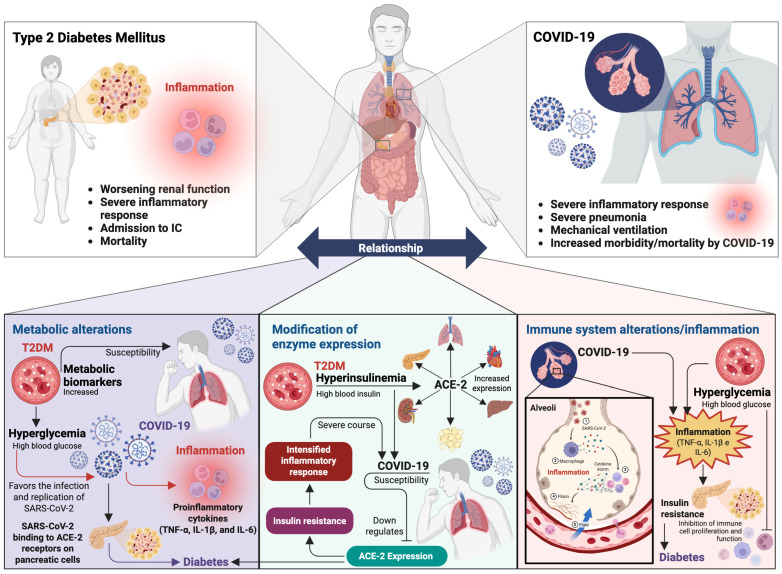
Interrelation between T2DM and COVID-19. The figure illustrates the fact that the interaction between both pathologies is mainly explained by three mechanisms. First mechanism: metabolic alterations, where hyperglycemia favors an environment conducive to the progression of the infection. Second mechanism: modification of the enzyme expression, such as ACE-2, a key receptor for SARS-CoV-2 entry into cells. Third mechanism: immune system dysfunction and increased systemic inflammation. These processes interrelate, promoting greater susceptibility to severe viral infections and exacerbating the complications associated with both diseases. Figure created with BioRender (https://www.biorender.com) by Muñoz-Carrillo et al.

Based on current scientific evidence, the following pathophysiological mechanisms are proposed, which could be associated with the comorbidity and susceptibility of patients with T2DM to adverse outcomes related to COVID-19, and which in turn are interrelated (Figure 4): (1) metabolic disorders; (2) modification of the enzyme expression related to SARS-CoV-2 infection; and (3) alterations of the immune system and inflammation.

*Metabolic alterations*. T2DM can increase the pathogenicity of the SARS-CoV-2 virus, partly due to metabolic alterations, increasing the susceptibility and severity of COVID-19, making patients with T2DM a high-risk population [212]. Studies have reported that in patients with T2DM, the increase in the plasma atherogenic index (AIP) [213], glucose [214,215], total cholesterol [216], triglycerides [217], and HbA1c [218] showed a significant positive correlation, indicating a susceptibility to COVID-19, because high levels of these metabolic factors increase the risk of contracting SARS-CoV-2 infection [219], associated with intubation and admission to intensive care in hospitalized infected patients [213]. Furthermore, chronic exposure to hyperglycemia also induces non-enzymatic changes in red blood cells, resulting in an increase in HbA1c [220,221]. Therefore, elevated levels of HbA1c have been significantly correlated with COVID-19 infection [222,223]. In this context, poor metabolic control in patients with T2DM is associated with increased morbidity and mortality from COVID-19 [224]. However, successful control of metabolism and blood glucose levels can effectively reduce severe disease progression and mortality [225] [226]. It has been reported that COVID-19, in patients with well-controlled T2DM, impacts at the metabolic level, since it has been observed that a mild or moderate SARS-CoV-2 infection decreases metabolic parameters, such as total cholesterol, triglycerides, body mass index and insulin-related indices, which could be related to the sustained effects of the acute phase of the infection [227].

Hyperglycemia plays an important role in the pathogenesis of T2DM, making patients more vulnerable to COVID-19 [228], since it can influence the expression of enzymes that favor the infection and replication of SARS-CoV-2 [229], as well as promoting the exacerbation of the inflammatory response through the production of proinflammatory cytokines (TNF-α, IL-1β, and IL-6) and the dysfunction of immune cells during the comorbidity of T2DM and COVID-19 [230,231,232]. Therefore, T2DM and its metabolic complications are associated with increased morbidity and mortality in patients with COVID-19 [233]. Interestingly, current scientific evidence reveals that when SARS-CoV-2 binds to ACE-2 on pancreatic cells, it can cause damage and reduce insulin production [234,235,236]. At the same time, the viral infection triggers an intense inflammatory response, characterized by an increase in inflammatory markers that can further damage pancreatic cells and lead to insulin resistance, which in turn increases blood glucose levels [237]. This process can lead to hyperglycemia in people without previous diabetes, worsen existing diabetes, or even trigger hyperglycemic emergencies [238]. In this regard, it has been observed that hospitalized patients with long-term COVID-19 have developed diabetes within one year of acute COVID-19 infection [239]. Therefore, it can be inferred that the presence of prolonged COVID-19 is a significant risk for an altered metabolic state, which can cause diabetes [240].

*Modification of the expression of enzymes related to SARS-CoV-2 infection*. ACE-2 plays an important role during the development of T2DM [241], since hyperinsulinemia increases its expression [242] in the lung, kidney, heart, liver, adipose tissue, and pancreas, which facilitates the entry of SARS-CoV-2 into cells and amplifies the viral burden [243,244,245]. Therefore, patients with T2DM are more vulnerable to COVID-19 [246]. However, SARS-CoV-2 infection decreases ACE-2 expression, which increases angiotensin II activity, leading to insulin resistance, a heightened immune response, and a severe course of COVID-19 [247]. On the other hand, the fact that patients with T2DM are more susceptible to COVID-19 is due to their inability to efficiently eliminate SARS-CoV-2. This is because patients with T2DM also express high levels of furin and TMPRSS-2, enzymes involved in the entry of the virus into the cell [248,249]. Furthermore, recent studies suggest that ACE-2, furin, and TMPRSS-2 are expressed in acinar, ductal, beta, alpha, mesenchymal, and endothelial cells in the pancreas [31]. This suggests that SARS-CoV-2 can infect the exocrine/endocrine pancreas and damage islet cells to cause acute diabetes [250,251,252].

*Immune system alterations and inflammation*. T2DM and its related metabolic disorders are mainly characterized by systemic inflammation. In this context, current evidence supports the idea that the inflammatory response is one of the key mechanisms linking COVID-19 with T2DM [233]. Therefore, glucose metabolism disorders during T2DM favor the progression of COVID-19 [253], since it has been observed that hyperglycemia aggravates the immune dysfunction of patients with T2DM and COVID-19 [254]. In this context, chronic inflammatory status [255] and elevated levels of proinflammatory cytokines can increase the intensity of the inflammatory response, leading to disease worsening and even multiple organ failure in patients with COVID-19 [229,256,257]. Hyperglycemia increases the production of proinflammatory mediators, such as IL-6, IL-1β, and TNF-α, which play a crucial role in the development of chronic inflammation and thus impair immune system function [258]. IL-1β is implicated in pancreatic β-cell damage [259], while TNF-α induces peripheral insulin resistance by altering insulin signaling through serine phosphorylation with the consequent development of T2DM [260]. It has been reported that patients with T2DM and COVID-19 have elevated levels of proinflammatory cytokines, such as IL-6 and TNF-α, as well as higher white blood cell counts (such as neutrophils), suggesting a heightened inflammatory response compared to patients without T2DM [209]. Likewise, hyperglycemia has also been associated with altered function of immune system cells [261]. Patients with T2DM present alterations in the immune system due to glycosylation, which inhibit PMN chemotaxis, phagocytosis, and intracellular destruction of pathogens, in addition to infecting macrophages and benefiting from the increase in the glycolytic rate in these cells, delaying the activation of Th1 cells and the hyperinflammatory response [262,263], increasing the risk of complications of COVID-19. On the other hand, patients with COVID-19 present low levels of peripheral CD4+ and CD8+ T lymphocytes. In this sense, hyperglycemia affects the immune response to COVID-19, inhibiting lymphocyte proliferation and weakening the role of immune cells, increasing the risk of severe clinical outcomes of COVID-19, such as pneumonia, in hospitalized patients with T2DM [257,264].

## 6. Discussion: Interrelationship Between Periodontitis, Type 2 Diabetes Mellitus, and COVID-19

Current scientific evidence on the interrelationship between periodontitis, T2DM, and COVID-19 is limited, with only two studies available that jointly address these three pathologies. Bachtiar et al. (2024) evaluated the association between dysbiotic periopathogens and inflammatory initiators and mediators in patients with COVID-19 and diabetes, and observed that patients with periodontitis, T2DM, and COVID-19 had a positive correlation (*p* < 0.05) between ACE-2 expression and inflammatory markers [265]. However, this study has some limitations, such as the impossibility of eliminating all confounding factors from an observational study design, the small sample size, and the use of relative abundance of microorganisms rather than absolute levels. Furthermore, a direct correlation between local and systemic inflammatory determinants could not be established. Nevertheless, the findings provide important information about the relationship between these diseases. On the other hand, a systematic review carried out by Casillas Santana et al. (2021) [266] hypothesizes that the relationship between these three pathologies is because T2DM is a metabolic disorder characterized by hyperglycemia in the blood, the result of altered secretion or action of insulin. Likewise, periodontitis and T2DM are inflammatory disorders with a bidirectional association, which share a similar immunomodulatory cascade and cytokine profile. On the other hand, ACE-2 is a crucial component of the renin–angiotensin system, and a key entry factor into SARS-CoV-2 cells. ACE-2 is widely distributed in various tissues including the oral cavity, mainly in the tongue and periodontal tissue. ACE-2 expression is modified by chronic uncontrolled glycemia in T2DM. Therefore, uncontrolled hyperglycemia increases the risk of developing periodontitis and triggers an overexpression of ACE-2 in the periodontal tissue of patients with T2DM, these events being potentially essential for SARS-CoV-2 infection and the development of the mild-to-severe form of COVID-19 [266]. However, this systematic review has certain limitations, particularly in its search strategy, as it only considered studies published in English, omitting studies in Spanish. Furthermore, it was conducted in 2021.

**Figure 5:**
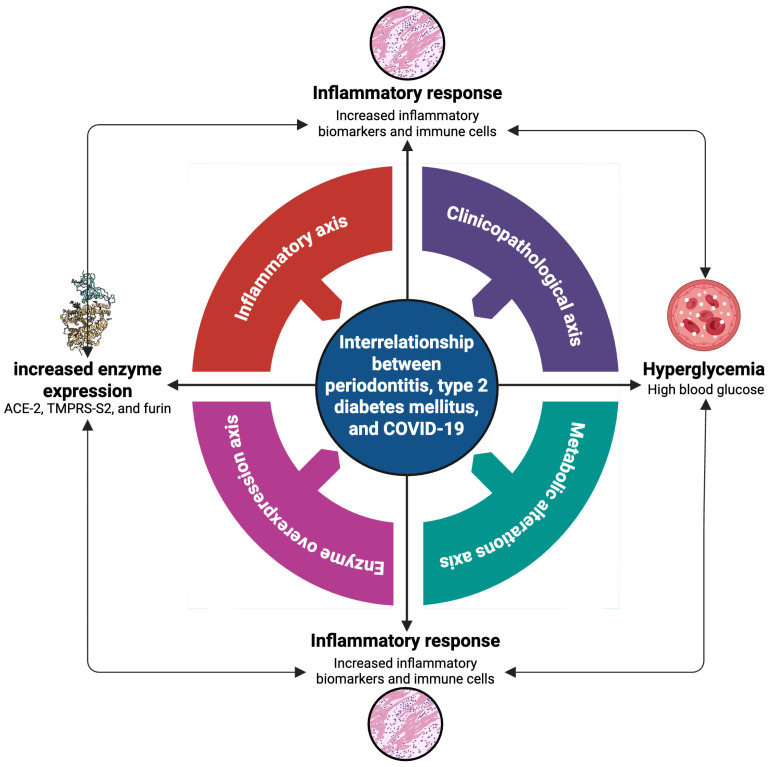
Interrelation between periodontitis, T2DM, and COVID-19. The figure illustrates the complex interplay between these three chronic conditions, which share four important axes: (1) a clinicopathological axis; (2) an axis associated with metabolic alterations; (3) an axis related to enzyme overexpression; and (4) an inflammatory axis. Figure created with BioRender (https://www.biorender.com) by Muñoz-Carrillo et al.

Although there is abundant information and scientific evidence on periodontitis, T2DM, and COVID-19, there is currently insufficient scientific literature addressing the relationship between these three diseases. Therefore, the main aim of this narrative review was to provide the current landscape of the interrelationship between periodontitis, T2DM, and COVID-19. In the present study, we sought to explain the interrelationship between the following comorbidities: (1) periodontitis and T2DM; (2) periodontitis and COVID-19; and (3) T2DM and COVID-19. Based on the reviewed literature, in addition to the few published scientific studies on the relationship between these three pathologies, we can hypothesize that the three diseases share four important axes (Figure 5): (1) a clinicopathological axis; (2) an axis associated with metabolic alterations; (3) an axis related to enzyme overexpression; and (4) an inflammatory axis.

*Clinicopathological axis*. Scientific research has consistently shown that patients with T2DM have a higher risk of developing periodontitis and often have more severe forms of the disease. Those patients with T2DM who do not adequately control their blood glucose levels are particularly prone to more severe periodontitis. This suggests a bidirectional relationship between T2DM and periodontal disease, as each condition can influence and aggravate the other [38,39,40,41,42,43]. Similarly, it has been observed that patients with periodontitis have a higher risk of suffering a more severe course of COVID-19 [117,118,119,120], including the need for hospitalization, mechanical ventilation, admission to intensive care, and a higher probability of death [126,127,128]. This is largely because both diseases are interrelated by systemic inflammatory mechanisms. COVID-19 can also worsen periodontal disease by disrupting the balance of periodontal–pathogenic bacteria, which can lead to infections, an exacerbated immune response, and increased inflammation. All of these factors together contribute to worsening periodontitis in patients with COVID-19 [131].

Regarding T2DM and COVID-19, clinical evidence has revealed an increased risk of serious complications when both diseases are present. Patients with this comorbidity (T2DM/COVID-19) tend to experience more severe respiratory problems, a higher incidence of acute complications, impaired kidney function, and worse overall outcomes. This is mainly due to an excessive inflammatory response, which significantly increases the risk of intensive care admission, and the need for mechanical ventilation, increasing the risk of death in hospitalized patients with T2DM and COVID-19 [202,203,204,205,206,207,208,209,210,211]. This clinicopathological axis highlights the interconnections between periodontitis, T2DM, and COVID-19, underscoring how these conditions influence each other mainly through inflammatory mechanisms, leading to an increased risk of severe complications and worse clinical outcomes for patients suffering from these comorbidities.

*Axis associated with metabolic alterations*. A key feature of T2DM is difficulty controlling blood glucose levels. When the disease is not adequately controlled, patients experience high glucose levels (hyperglycemia). This hyperglycemia causes various health problems, including an increased risk of developing comorbidities with other diseases [46]. In patients with T2DM, hyperglycemia triggers inflammatory processes [62,63,64,65] that can lead to insulin resistance [63], decreased endothelial function [90], an increase in PMN [47,48,49], and increased production of MMP [103,104]. These factors influence periodontitis, increasing the destruction of periodontal tissue due to an exacerbation of the inflammatory response, which leads to a more severe course of the disease [116]. In this regard, T2DM is considered a significant risk factor for the development and severity of periodontitis, and vice versa [57,58]. In this context, these conditions favor the infection capacity of SARS-CoV-2 [131]. Periodontal–pathogenic bacteria can reach the lungs through aspiration and cause overproduction of the ACE-2 in the alveoli [155]. This triggers lung inflammation and a massive release of inflammatory cytokines, generating a cytokine storm that induces damage to lung tissue [148,149,150,151]. This process is exacerbated in patients with T2DM, as hyperglycemia further intensifies the release of cytokines in the lungs. Furthermore, T2DM weakens the immune system, which favors the severity of COVID-19 disease [123,124,125]. This axis, associated with metabolic alterations, describes how T2DM, through hyperglycemia and inflammation, increases the risk and severity of periodontitis and COVID-19, indicating a strong interrelationship between the three diseases.

*Axis related to enzyme overexpression*. This axis describes the complex interaction between periodontitis, T2DM, and COVID-19, focusing on the crucial role of enzyme overexpression. ACE-2, present in several cells of the oral mucosa, including the tongue, gingival tissues, and salivary glands [145], acts as a key entry point for SARS-CoV-2 [146]. Surprisingly, ACE-2 expression is higher in salivary glands than in the lungs, making the oral cavity an important viral reservoir. Furthermore, periodontal–pathogenic bacteria such as *P. gingivalis* can increase the expression of ACE-2 and other molecules (TMPRSS-2 and furin) [155] that facilitate SARS-CoV-2 infection [156]. Hyperglycemia, a hallmark of T2DM, further exacerbates the situation by increasing vulnerability to COVID-19 [228], as it also influences the expression of these enzymes, favoring viral infection and exacerbating the inflammatory response, contributing to the severity of the disease [229,230,231,232]. Consequently, patients with T2DM are more susceptible to serious complications of COVID-19. Furthermore, SARS-CoV-2 can damage insulin-producing pancreatic cells, which can lead to hyperglycemia or diabetes, even in patients without the disease [234,235,236]. ACE-2 also plays a role in the development of T2DM [241], as hyperinsulinemia increases its expression in various organs [228], facilitating SARS-CoV-2 entry and amplifying viral burden. Consequently, patients with T2DM are more susceptible to severe complications of COVID-19 [246]. In summary, this axis describes a complex relationship between these three diseases, which mutually enhance each other through ACE-2 overexpression, inflammation, and altered glucose metabolism.

*Inflammatory axis*. The integration of this axis is even more complex, due to the interconnected pathways between periodontitis, T2DM, and COVID-19 disease. However, the common denominator within the axis is inflammation. Periodontitis is caused, mainly, by the inflammatory response induced by periodontal–pathogenic bacteria residing in dental plaque [54,55]. This inflammatory response is characterized by an increase in proinflammatory cytokines, such as TNF-α, IL-1β, and IL-6, and immune system cell populations [66]. In particular, the aberrant production of TNF-α, on the one hand, generates decreased vascular function [81]. On the other hand, it induces the increase and survival of PMN in the periodontal tissue [59,61], which in turn produces MMP-2, which leads to the destruction of periodontal tissue [116]. Likewise, TNF-α modulates the expression of endocan, a proteoglycan that acts as a proinflammatory mediator, which is associated with the most severe course of the disease [90]. Regarding IL-1β, this proinflammatory cytokine is associated with the activation of the inflammasome (such as overexpression of NLRP3), amplifying the inflammatory response and therefore the destruction of gingival tissue [101]. The intersection between periodontitis, T2DM, and COVID-19 disease occurs when, during diabetes mellitus, there is an increase in blood glucose levels (hyperglycemia) and, together with the viral infection, an exacerbated inflammatory response is triggered, increasing the production of TNF-α, IL-1β, IL-6, endocan, and NLRP3 inflammasome, and causing an increase in the PMN population, amplifying their effects and leading to a more severe course of comorbidity between these three pathologies [66,90,101]. In turn, during COVID-19 disease, periodontitis facilitates the passage of periodontal–pathogenic bacteria invading the lung, which increase the expression of ACE-2, favoring SARS-CoV-2 infection [131,146], which in turn produces a strong inflammatory response, also characterized by the aberrant production of proinflammatory cytokines (TNF-α, IL-1β, and IL-6) [145,166,172], and the activation of alveolar macrophages, which leads to a cytokine storm, which ultimately induces tissue destruction at the lung level, generating respiratory failure [170]. However, this cytokine storm manages to reach systemic circulation, which reaches the periodontal tissues, also favoring their destruction [162]. Likewise, T2DM and COVID-19 are related through systemic inflammation [233], where the hyperglycemia characteristic of T2DM worsens immune dysfunction and the inflammatory response in patients with COVID-19 [254], increasing the production of proinflammatory cytokines and impairing the function of immune cells [229,255,256,257]. This leads to an increased risk of serious complications, such as multiple organ failure and severe pneumonia, as hyperglycemia negatively affects chemotaxis, phagocytosis, and immune cell function, while COVID-19 depletes T cells, causing inflammation and immune dysfunction, which exacerbates the disease [224,262,263]. In this context, this axis describes how periodontitis, T2DM, and COVID-19 potentiate each other through inflammation. Periodontitis initiates inflammation in periodontal tissues, T2DM aggravates it with hyperglycemia, and COVID-19 facilitates the spread of oral bacteria to the lungs, causing severe inflammation. These three conditions create a relationship whose common axis is inflammation, which worsens the patient’s health.

## 7. Conclusions

In conclusion, this comprehensive narrative review reveals the complex network of interactions between periodontitis, T2DM, and COVID-19, with inflammation emerging as a key factor. The four interconnected axes—(1) a clinicopathological axis; (2) an axis associated with metabolic alterations; (3) an axis related to enzyme overexpression; and (4) an inflammatory axis—have been demonstrated to mutually enhance these three diseases, creating a “vicious cycle”, which worsens the patient’s health.

The clinicopathological axis highlights the bidirectional relationship between periodontitis and T2DM, with each exacerbating the other through systemic inflammatory mechanisms. It also highlights how COVID-19 contributes to this interaction, exacerbating both conditions and increasing the risk of serious complications. The metabolic alterations axis delves into the role of hyperglycemia, a hallmark of T2DM, as a key factor in exacerbating inflammation and increasing susceptibility to COVID-19. It describes how hyperglycemia triggers a cascade of events that promote periodontal tissue destruction and the severity of SARS-CoV-2 infection. The enzyme overexpression axis focuses on the crucial role of ACE-2 as an entry point for SARS-CoV-2, particularly in the oral cavity. It reveals how hyperglycemia and periodontal–pathogenic bacteria can increase ACE-2 expression, facilitating viral infection and exacerbating the inflammatory response. Together, these axes converge on a crucial axis: systemic inflammation. Inflammation, triggered by periodontitis, exacerbated by hyperglycemia in T2DM, and enhanced by SARS-CoV-2 infection, becomes the main mechanism linking these three conditions. This uncontrolled inflammation leads to an increased risk of serious complications, such as multiple organ failure, severe pneumonia, and mortality.

Currently, there is abundant information on these three diseases; however, our understanding of their complex interactions still presents significant gaps, underscoring the need for additional research to clarify this relationship. However, the available information already highlights the crucial importance of understanding these interconnections to develop comprehensive preventive and therapeutic strategies. To achieve this, a multidisciplinary approach bringing together healthcare professionals is required, thereby mitigating the impact of these comorbidities and improving patients’ quality of life.

## Data Availability

Not applicable.

## Abbreviations

The following abbreviations are used in this manuscript:

ACE-1	angiotensin-converting enzyme 1
ACE-2	angiotensin-converting enzyme 2
AIP	plasma atherogenic index
ARD	acute respiratory distress syndrome
CD147	cluster of differentiation 147
COVID-19	coronavirus disease 2019
ECM	extracellular matrix
GCF	gingival crevicular fluid
HbA1c	glycosylated hemoglobin
HGEC	human gingival epithelial cells
IL-6R	IL-6 receptor
IL	interleukin
iNOS	inducible nitric oxide synthase
LPS	lipopolysaccharide
MAPK	mitogen-activated protein kinase
MMP	matrix metalloproteinases
NET	neutrophil extracellular traps
NETosis	generation of NET
NF-κB	nuclear factor kappa B
NLR	NOD-like receptor
NLRP-3	NLR family pyrin domain containing 3
NO	nitric oxide
PGE_2_	prostaglandin E_2_
PMN	polymorphonuclear cells
RANKL	receptor activator of nuclear factor-κB ligand
SARS-CoV-2	severe acute respiratory syndrome coronavirus type 2
T2DM	type 2 diabetes mellitus
TGF-β	transforming growth factor beta
Th1 cells	type 1 T helper cells
TIMPs	tissue inhibitors of matrix metalloproteinases
TMPRSS-2	transmembrane serine protease 2
TNF-α	tumor necrosis factor alpha
